# Determination of quantitative and site-specific DNA methylation of *perforin *by pyrosequencing

**DOI:** 10.1186/1756-0500-2-104

**Published:** 2009-06-12

**Authors:** Supraja Narasimhan, Virginia R Falkenberg, Maung M Khin, Mangalathu S Rajeevan

**Affiliations:** 1 Division of Viral and Rickettsial Diseases, National Center for Zoonotic Vector-Borne and Enteric Diseases, Centers for Disease Control and Prevention, Atlanta, GA 30333, USA

## Abstract

**Background:**

Differential expression of perforin (*PRF1*), a gene with a pivotal role in immune surveillance, can be attributed to differential methylation of CpG sites in its promoter region. A reproducible method for quantitative and CpG site-specific determination of perforin methylation is required for molecular epidemiologic studies of chronic diseases with immune dysfunction.

**Findings:**

We developed a pyrosequencing based method to quantify site-specific methylation levels in 32 out of 34 CpG sites in the *PRF1 *promoter, and also compared methylation pattern in DNAs extracted from whole blood drawn into PAXgene blood DNA tubes (whole blood DNA) or DNA extracted from peripheral blood mononuclear cells (PBMC DNA) from the same normal subjects. Sodium bisulfite treatment of DNA and touchdown PCR were highly reproducible (coefficient of variation 1.63 to 2.18%) to preserve methylation information. Application of optimized pyrosequencing protocol to whole blood DNA revealed that methylation level varied along the promoter in normal subjects with extremely high methylation (mean 86%; range 82–92%) in the distal enhancer region (CpG sites 1–10), a variable methylation (range 49%–83%) in the methylation sensitive region (CpG sites 11–17), and a progressively declining methylation level (range 12%–80%) in the proximal promoter region (CpG sites 18–32) of *PRF1*. This pattern of methylation remained the same between whole blood and PBMC DNAs, but the absolute values of methylation in 30 out of 32 CpG sites differed significantly, with higher values for all CpG sites in the whole blood DNA.

**Conclusion:**

This reproducible, site-specific and quantitative method for methylation determination of *PRF1 *based on pyrosequencing without cloning is well suited for large-scale molecular epidemiologic studies of diseases with immune dysfunction. PBMC DNA may be better suited than whole blood DNA for examining methylation levels in genes associated with immune function.

## Background

Perforin (*PRF1*), a major immune surveillance gene, is a candidate gene for the identification of methylation markers, and for investigating the pathophysiology of a number of chronic diseases with suspected immune dysfunction [1-9]. Earlier studies to assess *PRF1 *promoter methylation used bisulfite sequencing of cloned PCR products [5]. Cloning PCR products for quantitative site-specific determination of CpG methylation is laborious, and can be inaccurate due to under-representation of the number of clones analyzed. On the other hand, pyrosequencing technology offers a convenient way for quantitative site-specific determination of CpG methylation but this technology has not yet been developed for the study of *PRF1*. Pyrosequencing determines the ratio of C/T base changes after bisulfite treatment, reflecting the proportion of unmethylated and methylated cytosines at each CpG site in the original sequence [10].

Several factors like sample collection, bisulfite treatment, and PCR can affect the reproducibility of methylation levels determined by pyrosequencing. Blood is a non-invasive source of sample for molecular epidemiologic studies. Usually, blood is drawn into blood collection tubes followed by separation of peripheral blood mononuclear cells (PBMCs) to extract DNA for molecular genetic studies including methylation. Recently, to minimize variability in primary blood samples from multicenter studies and to enable transport and storage, blood is drawn into specialized tubes like PAXgene blood DNA tubes containing additives for cell lysis. These PAXgene blood DNA tubes are then transported to laboratories where DNA is extracted directly from the whole blood without PBMC separation. DNA extracted from PAXgene blood DNA tubes thus represents the contribution of all blood cell types and have been successfully used for genotyping. However, the impact of these blood collection procedures for DNA (whole blood vs PBMC DNA) on methylation levels has not been reported.

In this study, we developed a pyrosequencing based methylation assay for a total of 32 out of 34 CpG sites in the promoter region of *PRF1*. The assay is highly reproducible with respect to bisulfite treatment, and PCR. Examination of methylation levels in normal subjects showed that methylation levels differ significantly between DNAs extracted from PBMCs and whole blood for all 32 CpG sites except two, although the methylation pattern remained similar over the 1.4 kb promoter region. These results show that site-specific assessment of CpG methylation in *PRF1 *can be reproducibly assessed by pyrosequencing without cloning, and that PBMC DNA may be better suited than whole blood DNA for molecular epidemiologic studies on methylation levels of genes associated with immune dysfunction.

## Methods

### Pyrosequencing strategy

Bisulfite converted sequences were simulated by a previously reported Microsoft Word bisulfite macro [11] followed by pyrosequencing assay design using Assay Design Software (Biotage, Uppsala, Sweden). Both top and bottom strands were used for designing primers to cover all the 34 CpG sites in 1.4 kb *PRF1 *promoter. Similarly, both forward and reverse sequencing designs were employed with 5'biotinylation of the reverse or forward PCR primer respectively. All primers were synthesized at the Biotechnology Core Facility, Centers for Disease Control & Prevention, Atlanta, GA.

### DNA samples and bisulfite treatment

Blood drawn in PAXgene blood DNA tubes (Qiagen, CA) for DNA extraction were obtained from 20 subjects who took part in the Chronic Fatigue Syndrome Study at the Centers for Disease Control and Prevention, Atlanta, GA. From five of these subjects classified as normal, blood was also drawn in BD Vacutainer CPT tubes (BD, NJ) followed by separation of PBMC for DNA extraction. DNA from PAXgene blood tubes was extracted using PAXgene blood DNA kit (Qiagen) whereas DNA from PBMCs was extracted using Roche DNA isolation kit for mammalian blood (Roche Applied Science, IN). DNA was quantified using NanoDrop ND-100 Spectrophotometer (Thermo Fisher Scientific, MA) prior to bisulfite treatment. DNA (200 ng/reaction) was bisulfite treated using the EZ DNA Methylation Kit (Zymo Research, CA), according to the manufacturer's instructions. Bisulfite treated DNA (BST-DNA) was eluted twice in 10 μl of the manufacturer's M-Elution Buffer (20 μl final volume), and was stored in aliquots at -20°C until use.

### PCR and pyrosequencing

Each 100 μl PCR contained 1.5 mM MgCl_2_, 0.2 mM dNTP, 0.05 U/μl Platinum Taq DNA polymerase (Invitrogen, CA) and 0.2 μM each of forward and reverse primers (Table 1) and 5 μl of BST-DNA. In this study, we used a touchdown PCR cycling approach previously reported for BST-DNA [12] for all amplicons except amplicon F (Table 1). Touchdown PCR consisted of one cycle of 94°C for 5 min for the initial denaturation step. This was followed by 5 cycles each of denaturation at 94°C for 30 s, varying annealing for 30 s, and extension at 72°C for 30 s. Annealing temperatures for the touchdown portion were provided as follows: 65°C for 5 cycles, 62°C for 5 cycles, 59°C for 5 cycles, 56°C for 5 cycles and 52°C for 5 cycles. A further 50 cycles constituted the following: 94°C for 30 s, 50°C for 30 s and 72°C for 30 s. PCR was terminated after a final cycle at 72°C for 7 min. PCR cycling conditions for amplicon F consisted of the following: one cycle of 95°C for 2 min; 50 cycles of 95°C for 15 s, 52°C for 30 s, 72°C for 30 s; one cycle of 72°C for 5 min.

**Table 1:** Primer sets for determination of *perforin *CpG methylation by pyrosequencing

Amplicon* (bp)	PCR primers^† ^(5' to 3')	Sequencing primers^‡^	CpG site # and nucleotide position^∞^
A (236)	FW: TTG ATT TTA TAG GTG AGG AAA TTA RV: **TTC CAA CTA TCA CCC ATA ACC TAA**	**TTT TAT AGG TGA GGA AAT TA** *AYGTTTAGAAAGGGGGTTGATATTTATYGTYGTGAGGTATAYGG* **ATT TTT ATG TTT TTT AAA T** *YGGTTTTTTTGTTA*	1, -1365; 2, -1339; 3, -1336; 4, -1325 6, -1231

**B **(226)	FW: ATG TTG AGG TTG TGA GGA GTT TT RV: **AAA TTC CAA AAT CCT CTC TTT AAT**	**GAA GTT TTG TAA AGT ATT TG** *YGGGAAAAGAG*	5, -1267

C (196)	FW: TTG GAA AGT GAT TAG GAG GTT GTA RV: **CAA ACT CCA AAC CAC ATA TAA CAT**	**AGA GGG TGG GGA TATT** *GYGGAGAGAAGATGGGGTTAGATTTYG* **GTT TTT GTT TTT GTA AGA GT** *AGGGAYGGAAGTAGGGATATAAAYG*	7, -1110; 8, -1088 9, -1045; 10, -1027

**D **(91)	FW: GAG GTT TTT ATG GGT GGA GTG AT RV:**CAC CTC CTC CCT TAC CCA ACT A**	**TTG GGG GGT AAA ATT** *ATAYGGTTTT T*	11, -876

**E **(256)	FW: TTT TGA GTG GGA GAA GAG AGA TGT RV:**CCC CAC CCT AAC CTC AAA CA**	**GTG AGA GTG GTT TGG TAG** *TATYGGAGG* **TTG GTT TTA GTT TTG TTG A** *GGTYGTGGGT* **AGG ATA GTT AGT GGT TTT TA** *YGTTGGTTTTAGTTTTGTTG*	12, -776 13, -774 14, -720

F (167)	FW: TGG AGG TTA TTG GTT GTT TTT ATA RV: **TAA CCA TTC CCT CCT CCC TAA ATA**	**GGT TAT TGG TTG TTT TTA TA** *AAGYGAGGAGTAGGAGTTTTTGTTYGAGGAATATGTTTGGAGTTYGG*	15, -691; 16, -670; 17, -650

G (300)	FW: TTA GGG AGG AGG GAA TGG TTA TAG RV: **AAC CAA CAA AAC CAT CTC CTT ACT**	**TAG TTT ATA TTG TTG GTG TA** *TAATYGAGTTGTTTAAGTTTYGGYGGTTTGGYG*	18, -397; 19, -381; 20, -378; 21, -370

H (300)	FW: **TTT AGG GAG GAG GGA ATG GTT ATA** RV: ACC AAC AAA ACC ATC TCC TTA CTT	**TAC TTC TAA TAC ACA ACA TC** *RCATATATAAAATATAAAAAACAAACAAAAACRACRAC*	24, -313; 23, -345; 22, -348

**I **(233)	FW: GGG GAT TTA GGG TAT ATA GG RV: **AAA CCC TAC CAA TCC ACA CTA CT**	**GGG GAT TTA GGG TAT AT** *AGGYGGAGGAGGGYGGGGYGTTGAGGATTTTGAGATTYGGT*	28, -219; 27, -229; 26, -234; 25, -253

J (237)	FW: **TGG TTT TGT TGG TTT GTT TAT TAA** RV: CCC CAA CTA TAA TCA CAA ATC CTT	**CAA AAC CAA AAA CTC ATC T** *ACCRAATAAAACTACTAAAACTCR* **CCT CAA CCC TCA TCC** *RACTCCCCACTAACAACCCTCAAAAAACRAAC*	30, -180; 29, -200 32, -122; 31, -150

K (97)	FW: TTG AGG ATA GGG TGG GTG TT RV: **CCA CCA CTC ACA TCA CTT CTA CTT**	**GGA TAG GGT GGG TGT T** *YGTGGGAGGGGAGAGTATAAAGGATTTGTGATTATAGTTGGGGGY*	33, -92; 34, -48

*Nucleotide positions of amplicons from 5' → 3' direction of the top or bottom strand used for assay design. A, 20 – 255; **B**, 1186 – 1411; C, 250 – 445; **D**, 826 – 916; **E**, 569 – 824; **F**, 692 – 858; G, 838 – 1137; H, 837 – 1136; **I**, 200 – 432; J, 1124 – 1360; K, 1298 – 1394. Amplicons in bold (B, D, E and I) represent those amplicons designed using the bottom strand after bisulfite conversion.

^†^Fw, forward primer; Rv, reverse primer. Biotinylated primers are indicated in bold.

^†^Sequencing primers are indicated in bold. Sequences to analyze are italicized with the CpG sites (*Y *or *R*) underlined.

^∞^Indicates CpG site number and corresponding nucleotide location from the distal end of the promoter. Numbering is based on the top strand used for assay design.

Pyrosequencing was performed according to the manufacturer's instructions. The PyroGold kit was used in conjunction with the PSQ 96MA instrument (Biotage), and each pyrosequencing reaction used 20 μl of PCR product. Biotinylated single strand DNA was separated by immobilizing the PCR product on streptavidin coated Sepharose High Performance beads (Amersham Biosciences, NJ) and strand separated by denaturation with 0.2 N NaOH. After denaturation, the biotinylated strand was annealed to sequencing primer (0.5 μM final concentration), and subjected to sequencing using an automatically generated nucleotide dispensation order for the "sequence to analyze" corresponding to each reaction (Table 1). Pyrograms were analyzed in "Allele Quantification" mode in order to determine the percentage of C/T, corresponding to the percentages of methylated and unmethylated C at each of the CpG sites. As a positive control for PCR and pyrosequencing, we used previously reported PCR and sequencing primers to detect two CpG sites in *CDNK2A *[13].

### Assay reproducibility

#### 1. Bisulfite reproducibility

The impact of bisulfite treatment on assay reproducibility was tested using the PCR and pyrosequencing conditions for CpG sites 18–21 in amplicon G with whole blood DNA from 20 subjects. The same DNA was bisulfite treated on three separate days. Each day, 20 individual bisulfite reactions were set up with 200 ng DNA/reaction. BST-DNA from each of the three days was simultaneously subjected to PCR in the same thermocycler to eliminate PCR-to-PCR variability. All PCR products were used for pyrosequencing in duplicate, giving a mean of 6 pyrogram readings per CpG site for all three sets of BST-DNA per subject. Day-to-day variation in bisulfite reaction was expressed as coefficient of variation (CV) calculated as the percentage of standard deviation to its mean

#### 2. Touchdown PCR reproducibility

The impact of PCR-to-PCR variability, expressed in terms of CV, was also tested for CpG sites 18–21 in amplicon G on three different days using the same BST-DNA. Touchdown PCR reproducibility was done with whole blood DNA from 10 subjects. Each PCR product was subjected to pyrosequencing in duplicate, yielding a mean of 6 values per CpG site for PCR from all three days per subject.

### Evaluation of methylation pattern in the PRF1 by pyrosequencing

A small pilot study consisting of five normal subjects was conducted to evaluate the robustness of the optimized conditions to determine the methylation level/pattern in the *PRF1 *promoter for 32 CpG sites. This pilot study also examined the impact of DNA from whole blood or PBMC on *PRF1 *methylation profile in normal subjects. For each subject, two separate bisulfite reactions were set up, followed by corresponding PCR and pyrosequencing reactions yielding a mean of 2 values per CpG site. Both whole blood and PBMC DNAs were treated similarly to generate the *PRF1 *methylation profiles.

## Results

**Figure 1:**
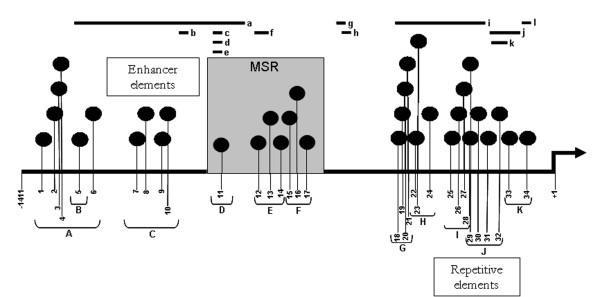
**The *perforin (PRF1) *promoter (1411 bp) with locations of 34 CpG and several putative transcription factor binding sites**. Ten CpGs are located in the enhancer element region, seven in the methylation-sensitive region (MSR) and 17 in the repetitive element region. "+1" indicates the transcription start site. Lower case letters from 'a -to- l' represent different transcription factor binding sites [14,15]: a, inducer response motif; b, γ-IFN responsive element; c, CRE element; d, AP-2 element; e, TPA-responsive element; f, STAT5-responsive enhancer; g, CCAT box; h, C-fos enhancer; i, 19 homologous repeats; j, three repeats; k, two repeats; l, GC box. Upper case letters from 'A -to- K' represent amplicons (not scaled to size) designed to detect different CpG sites.

The *PRF1 *promoter spans about 1.4 kb (-1411 to +1) upstream of the transcriptional start site and harbors a number of transcription factor binding sites [14,15]. Out of a total of 34 CpG sites in the *PRF1 *promoter, 10 are in the distal promoter region containing enhancer elements (CpG sites 1–10), 17 are in the proximal promoter region harboring repetitive elements (CpG sites 18–34), and seven (CpG sites 11–17) are in the methylation sensitive region (MSR) located between the distal and proximal promoter regions (Figure 1). Eleven amplicons A to K and 16 sequencing primers were designed to cover all 34 CpG sites (Figure 1; Table 1).

We compared the performance of PCR with single annealing temperature and touchdown PCR and found that PCR cycling program with a single annealing temperature often generated multiple bands, particularly with amplicon E, which is rich in AT content (70%). On the other hand, touchdown PCR consistently generated specific products of expected size with all amplicons except amplicon F, for which PCR with single annealing temperature consistently resulted in specific product than touchdown PCR. Thus, we used touchdown PCR to generate all amplicons, except amplicon F which was generated with PCR following single annealing temperature. Pyrosequencing reactions for all CpG sites were successful except CpG sites 33 and 34 in amplicon K, although amplicon K was successfully generated by the touchdown PCR.

**Figure 2:**
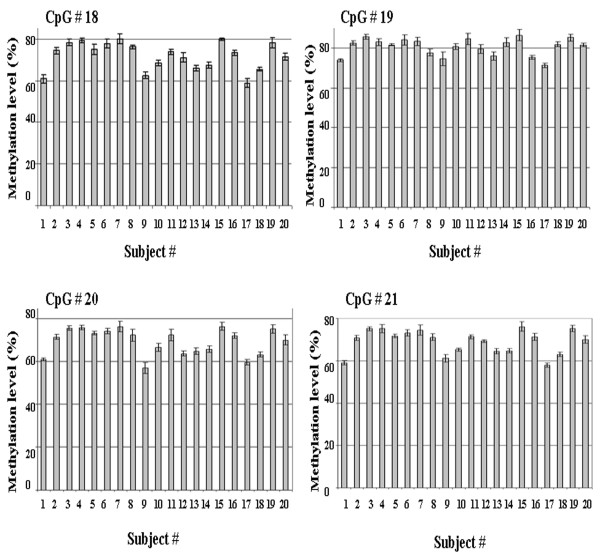
**Reproducibility of bisulfite treatment determined using amplicon G**. Mean methylation level (%) ± SD for four CpG sites 18–21 are shown.

**Figure 3:**
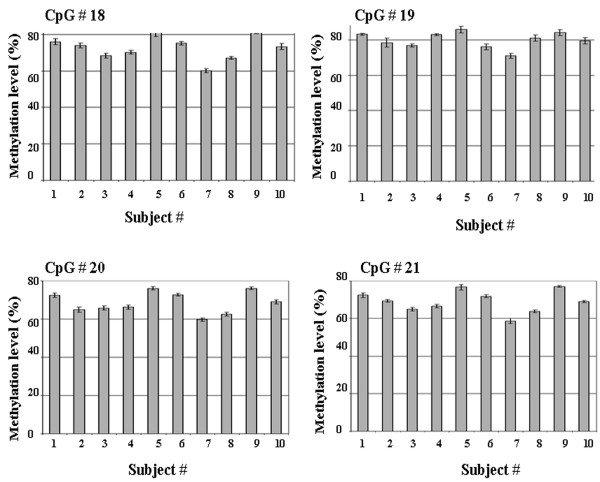
**Reproducibility of touchdown PCR determined using amplicon G**. Mean methylation level (%) ± SD for four CpG sites 18 to 21 are shown.

Variability due to bisulfite treatment was tested for four CpG sites (sites 18–21) in amplicon G using whole blood DNA from 20 individual subjects. Variability due to bisulfite treatment was found to be minimal for all four CpG sites in amplicon G, (mean CV of 2.18%, and CVs for individual CpG sites ranged from 1.97% to 2.37%, Figure 2). Variability due to touchdown PCR was also assessed for CPG sites 18–21 using whole blood DNA from 10 subjects and was found to be very low for all four CpG sites in amplicon G, with a mean CV of 1.63%, and with CVs for individual CpG sites ranging from 1.34% to 1.86% (Figure 3).

**Figure 4:**
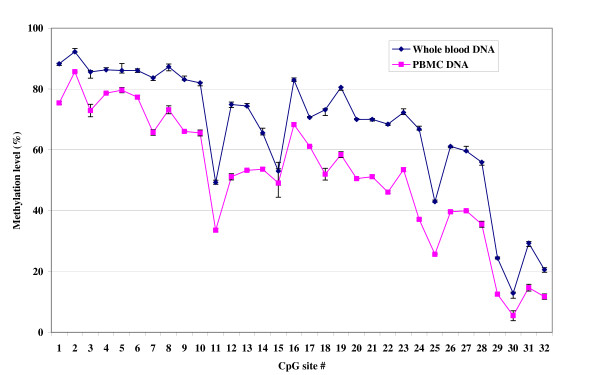
**The pattern of methylation in 32 CpG sites in 1.4 kb *PRF1 *promoter using DNA from whole blood and PBMCs from normal subjects**. Y-axis, methylation level (%) ± SD. X-axis, CpG sites 1–32. Methylation levels at each CpG site represent mean of 5 normal subjects.

Application of the optimized conditions to generate *PRF1 *methylation profile for 32 CpG sites using whole blood DNA from five normal subjects revealed that the methylation levels varied along the promoter with extremely high methylation (mean 86%; range 82%–92%) in the distal enhancer region covering CpG sites 1–10 (Figure 4). Methylation levels in the MSR covering CpG sites 11–17 appeared to vary up and down from a low of 49% (CV = 1.49%) at CpG site 11 to a high of 83% (CV = 0.84%) at CpG site 16 (mean methylation level for sites 11–17 = 67%). Interestingly, methylation levels in the proximal promoter region covering CpG sites 18–32 progressively declined from a high of 73–80% at sites 18–19 to as low as 12–20% at sites 30 and 32 (mean methylation at sites 18–32 = 54%). Among specific sites, CpG sites 11, 15, 25, 29 and 30 showed sharp decline in methylation levels compared to adjacent CpG sites. Perforin methylation profile over the same 32 CpG sites in the same five subjects using PBMC DNA appeared very similar i.e., extremely high methylation in the distal enhancer region, a variable methylation in the MSR, and progressively declining methylation in the proximal promoter region. However, except for CpG sites 5 and 15, the absolute methylation levels at each CpG site as determined from the whole blood and PBMC DNAs differed significantly, with higher values for all CpG sites in the whole blood DNA (Figure 4).

## Discussion

Analytical procedures for sensitive and site-specific quantitative determination of CpG methylation are influenced by several factors, all of which require careful experimental optimization and validation before the assay can be applied to valuable, and often limited clinical samples in large-scale molecular epidemiologic studies. A number of PCR-based methylation assays utilize bisulfite treatment to preserve the methylation signature of the original DNA. Bisulfite treatment selectively converts unmethylated cytosines to uracil. This base modification results in reduced sequence complexity, which often makes the development of methylation assays difficult due to diminished flexibility in designing quality PCR and sequencing primers, allele-specific PCR biases, and reduced signal strength or abnormally shaped sequencing reactions [16,17]. We considered these factors while developing a pyrosequencing assay to determine the methylation status of *PRF1*, an important gene in immune surveillance.

Pyrosequencing assay was designed for all 34 CpG sites in the *PRF1 *promoter, of which seven CpG sites form a functionally significant subset in the MSR of the promoter. We successfully optimized several steps of the pyrosequencing technology to obtain highly reproducible methylation levels for each of the 32 out of 34 CpG sites. Bisulfite treatment resulted in minimal day-to-day variation (CV 2.18%) that was comparable to the minimal reaction-to-reaction variation in bisulfite treatment as determined by fluorescent probe based quantitative version of methylation-sensitive PCR (MSP, MethyLight assay) [18]. A touchdown PCR approach has been recommended for overcoming PCR biases in reactions using BST-DNA template [12], but the reproducibility of touchdown PCR has not been examined with BST-DNA. We found that touchdown PCR helped to overcome problems with PCR product specificity, and resulted in highly reproducible results from PCR-to-PCR on different days (CV 1.63%).

In this study, the *PRF1 *methylation pattern identified using both whole blood and PBMC DNAs from normal subjects appeared similar throughout the promoter region in terms of extremely high methylation in distal enhancer region, variable methylation in the MSR, and progressively declining methylation in the proximal promoter region. Except CpG sites 5 and 15, the absolute levels of methylation at each of these CpG sites, however, differed between whole blood and PBMC DNAs, with higher methylation levels obtained using whole blood DNA. We have not investigated the factors that contribute to this similar pattern but different levels in methylation along the *PRF1 *promoter between whole blood and PBMC DNAs but it is likely to be related to differences in cell types between these samples from the same subject. In a previous study that used bisulfite sequencing followed by cloning, CpG site 14 was found to be the most highly methylated (> 50%) in the MSR of CD4^+ ^T and CD8^+ ^T cells of normal subjects [5]. On the contrary, the most highly methylated CpG site in the MSR in normal subjects in this study was found to be CpG site 16 located 70 bases away from CpG site 14. These differences may be related to differences in the subjects, cell populations or quantitative nature of the assays employed between the studies. The pysosequencing assay described in this report is highly suitable to quantify differences in the methylation of *PRF1 *in the various sub-populations of cells in blood from normal and subjects with chronic diseases of immune dysfunction. Further, the study suggests that blood drawn in PAXgene blood DNA tubes may be inappropriate for DNA methylation studies of genes with immune function.

We have not compared the estimates of *PRF1 *methylation determined by pyrosequencing with other method such as MSP or combined bisulfite restriction analysis since our reproducibility data agree with other reports supporting the reliability of pyrosequencing for quantitative and efficient screening of multiple CpG sites in candidate genes studies [13,19-21]. Further, pyrosequencing has been used as a reference method for validation of other methods and has been shown to have excellent specificity and sensitivity for diagnostic uses [22,23]. A number of dilution and titration experiments with known amounts of methylated and unmethylated DNA or cells with methylated viral DNA in the background of uninfected cells also support the reliability of pyrosequencing for the sensitive and specific determination of methylation at multiple CpG sites [10,13,18,23,24].

## Conclusion

In conclusion, we have developed a highly reproducible procedure based on pyrosequencing technology for the site-specific and quantitative determination of methylation levels in 32 CpG sites in the *PRF1 *promoter. In normal subjects, methylation levels along the 1.4 kb promoter varied similarly in DNAs from whole blood and PBMC but differed in absolute methylation levels, suggesting collection of blood in devices like PAXgene blood DNA tube may not be appropriate for methylation studies of immune function related genes. This method can be applied to clinical samples in order to systematically evaluate the significance of multiple CpG sites in perforin which will improve our understanding of the pathophysiology of chronic disorders with alterations in immune function.

## Competing interests

The authors declare that they have no competing interests.

## Authors' contributions

SN developed the pyrosequencing strategy, carried out the experiments and participated in manuscript writing. VRF contributed to experimental designs, interpretation of results and participated in the manuscript writing. MMK participated in the design, implementation of laboratory experiments, and manuscript writing. MSR conceived of this study and as Principle Investigator participated in all aspects of this study and drafting of this manuscript. All authors have read and approved the manuscript.

## References

[B1] Gulan G, Ravlic-Gulan J, Strbo N, Sotosek V, Nemec B, Matovinovic D (2003). Systemic and local expression of perforin in lymphocyte subsets in acute and chronic rheumatoid arthritis. J Rheumatol.

[B2] Kaplan MJ, Lu Q, Wu A, Attwood J, Richardson B (2004). Demethylation of promoter regulatory elements contributes to perforin overexpression in CD4+ lupus T cells. J Immunol.

[B3] Kastelan M, Prpic ML, Gruber F, Zamolo G, Zauhar G, Coklo M (2004). Perforin expression is upregulated in the epidermis of psoriatic lesions. Br J Dermatol.

[B4] Li M, Yang Q, Zhang Y (2007). Effects of CD134 monoclonal antibody on hemolysis activities and expression of perforin in peripheral blood mononuclear cells of systemic lupus erythematosus patients. Hybridoma (Larchmt).

[B5] Lu Q, Wu A, Ray D, Deng C, Attwood J, Hanash S (2003). DNA methylation and chromatin structure regulate T cell perforin gene expression. J Immunol.

[B6] Maher KJ, Klimas NG, Fletcher MA (2005). Chronic fatigue syndrome is associated with diminished intracellular perforin. Clin Exp Immunol.

[B7] Skarpa I, Rubesa G, Moro L, Manestar D, Petrovecki M, Rukavina D (2001). Changes of cytolytic cells and perforin expression in patients with posttraumatic stress disorder. Croat Med J.

[B8] Steinau M, Unger ER, Vernon SD, Jones JF, Rajeevan MS (2004). Differential-display PCR of peripheral blood for biomarker discovery in chronic fatigue syndrome. J Mol Med.

[B9] Trapani JA, Voskoboinik I (2007). The complex issue of regulating perforin expression. Trends Immunol.

[B10] Rajeevan MS, Swan DC, Duncan K, Lee DR, Limor JR, Unger ER (2006). Quantitation of site-specific HPV 16 DNA methylation by pyrosequencing. J Virol Methods.

[B11] Singal R, Grimes SR (2001). Microsoft Word macro for analysis of cytosine methylation by the bisulfite deamination reaction. Biotechniques.

[B12] Shen L, Guo Y, Chen X, Ahmed S, Issa JP (2007). Optimizing annealing temperature overcomes bias in bisulfite PCR methylation analysis. Biotechniques.

[B13] Colella S, Shen L, Baggerly KA, Issa JP, Krahe R (2003). Sensitive and quantitative universal Pyrosequencing methylation analysis of CpG sites. Biotechniques.

[B14] Lichtenheld MG, Podack ER (1989). Structure of the human perforin gene. A simple gene organization with interesting potential regulatory sequences. J Immunol.

[B15] Zhang J, Scordi I, Smyth MJ, Lichtenheld MG (1999). Interleukin 2 receptor signaling regulates the perforin gene through signal transducer and activator of transcription (Stat)5 activation of two enhancers. J Exp Med.

[B16] Gharizadeh B, Akhras M, Nourizad N, Ghaderi M, Yasuda K, Nyren P (2006). Methodological improvements of pyrosequencing technology. J Biotechnol.

[B17] Mashayekhi F, Ronaghi M (2007). Analysis of read length limiting factors in Pyrosequencing chemistry. Anal Biochem.

[B18] Ogino S, Kawasaki T, Brahmandam M, Cantor M, Kirkner GJ, Spiegelman D (2006). Precision and performance characteristics of bisulfite conversion and real-time PCR (MethyLight) for quantitative DNA methylation analysis. J Mol Diagn.

[B19] Dupont JM, Tost J, Jammes H, Gut IG (2004). De novo quantitative bisulfite sequencing using the pyrosequencing technology. Anal Biochem.

[B20] Mikeska T, Bock C, El-Maarri O, Hubner A, Ehrentraut D, Schramm J (2007). Optimization of quantitative MGMT promoter methylation analysis using pyrosequencing and combined bisulfite restriction analysis. J Mol Diagn.

[B21] Tost J, Gut IG (2007). DNA methylation analysis by pyrosequencing. Nat Protoc.

[B22] Irizarry RA, Ladd-Acosta C, Carvalho B, Wu H, Brandenburg SA, Jeddeloh JA (2008). Comprehensive high-throughput arrays for relative methylation (CHARM). Genome Res.

[B23] White HE, Durston VJ, Harvey JF, Cross NC (2006). Quantitative analysis of SNRPN(correction of SRNPN) gene methylation by pyrosequencing as a diagnostic test for Prader-Willi syndrome and Angelman syndrome. Clin Chem.

[B24] Uhlmann K, Brinckmann A, Toliat MR, Ritter H, Nurnberg P (2002). Evaluation of a potential epigenetic biomarker by quantitative methyl-single nucleotide polymorphism analysis. Electrophoresis.

